# Functionally Informed Hand Knob Reveals Structural Connectome Differences in Motor-Eloquent Tumours

**DOI:** 10.3390/cancers18182925

**Published:** 2026-09-09

**Authors:** Sankhya Prakashvel, Filippo Sinosi, Laura Ferrari, Feras Fayez, Sabina Patel, Yasir A. Chowdhury, Andrea Perera, Nida Kalyal, Mariam Awan, Alba Diaz-Baamonde, Ana Mirallave-Pescador, Keyoumars Ashkan, Ranjeev Bhangoo, Francesco Vergani, Jose Pedro Lavrador

**Affiliations:** 1 Queens’ Hospital Romford, London RM7 0AG, UK; 2 King’s College Hospital, London SE5 9RS, UK; filippo.sinusi@nhs.net (F.S.); spatel21@nhs.net (S.P.); yasir.chowdhury2@nhs.net (Y.A.C.); andrea.perera@nhs.net (A.P.); nida.kalyal@nhs.net (N.K.); m.awan1@nhs.net (M.A.); a.diazbaamonde@nhs.net (A.D.-B.); a.mirallave-pescador@nhs.net (A.M.-P.); k.ashkan@nhs.net (K.A.); ranj.bhangoo@nhs.net (R.B.); francesco.vergani@nhs.net (F.V.); 3 University College London Hospital, London NW1 2BU, UK; feras.fayez@nhs.net

**Keywords:** brain tumour, structural connectome, diffusion spectrum imaging, subcortical hyper-connectivity, basal ganglia, transcranial magnetic stimulation, IDH mutation, glioblastoma

## Abstract

**Simple Summary:**

When comparing the cortico-spinal tract between the grossly affected and unaffected hemispheres in the supratentorial compartment, tumour presence significantly reduced cortico-spinal tract volume and prolonged the cortical silent period (indicating intracortical inhibition) when compared to the unaffected hemisphere. In addition, structural connectomes with regard to the cortico-spinal tract in motor-eloquent brain tumours have been shown to maintain the global network topology in the cortices but alter and induce hyper-connectivity in the subcortices and especially in the basal ganglia motor loop. IDH mutant and slow-growing tumours were found to generate significantly more organized hyperconnected networks than the IDH-wildtype tumours.

**Abstract:**

**Background:** Brain tumours impose complex, spatially heterogeneous disturbances on neural circuits that extend far beyond the immediate lesion site. While gross anatomical displacement of the cortico-spinal tract (CST) has been extensively studied, the topological reorganization of the functionally informed structural connectome—and its dependence on tumour molecular phenotype—remains incompletely understood. **Objectives:** This study aimed to characterize upper-limb functionally informed network topology in brain tumour patients, identify histological and molecular patterns of structural reorganization at the cortical and subcortical level, and determine the impact on neurophysiological parameters. **Methods:** Forty-eight patients with supratentorial motor-eloquent tumours (MET’s) underwent diffusion-weighted imaging (DWI) as part of their preoperative motor mapping. Connectivity matrices based on streamline passing counts were extracted from 426 nodes of the HCPex atlas using DSI Studio^®^ upon seeding the structural connectome in the motor hotspot (best motor response) for the functional area of the upper limb identified using preoperative navigated transcranial magnetic stimulation (nTMS). Paired-sample *t*-tests compared tumour versus healthy hemispheres across network topology metrics. The impact of nTMS-derived excitability metrics—interhemispheric resting motor threshold ratio (iRMTr) and cortical silent period (CSP)—and tumour histological and molecular characteristics on the connectome was assessed. **Results:** The global network topology of the tumour hemisphere was preserved when compared to the healthy baseline hemisphere across all tumour types (*p* > 0.05). Subcortical analysis revealed significant hyper-connectivity in the tumour hemisphere, with elevated degree, strength, clustering coefficient, local efficiency, and eigenvector centrality (*p* < 0.05). Basal ganglia motor loop degree was increased in the tumour hemisphere (mean 7.37 *versus* 5.19; *p* = 0.0009). IDH-mutant tumours generated significantly more topologically organized compensatory networks than IDH-wildtype tumours (Clustering Coefficient 0.345 *versus* 0.273; *p* = 0.018). Of the cortical nodes on the side of the tumour, significant hyper-connectivity was seen in the supplementary motor area (*p* = 0.0011), premotor cortex (Area 6), with increased connection seen in 6 mp (medial premotor at *p* =0.0033) and 6 d (dorsal premotor at *p* = 0.025) and primary somatosensory cortex (*p* = 0.027). The presence of the tumour induced significant changes across all three domains: loss of CST volume (*p* < 0.001), prolongation of the cortical silent period indicative of intracortical inhibition (*p* < 0.0001), with significant prolongation in glioblastoma versus an oligodendroglioma. **Conclusions:** Brain tumours significantly impact the upper limb-centred structural connectome. While global network topology is preserved, tumours induce substantial subcortical and basal ganglia network reorganization by inducing compensatory hyper-connectivity. These findings suggest that structural connectomics offers a novel framework for non-invasive tumour characterization and surgical planning.

## 1. Introduction

Brain tumours impose substantial neurological burdens whose complexity extends well beyond the lesion’s anatomical footprint. Despite advances in surgery, radiotherapy, and systemic treatment, high-grade gliomas still carry a median survival of 14 to 16 months [1], and a central challenge in their management remains the incomplete understanding of how the tumour reshapes the surrounding neural architecture over time. Structural MRI describes the lesion itself but not the network-level consequences of its presence.

The cortico-spinal tract (CST) is the critical efferent structure at risk in supratentorial tumours, and its preservation is a primary surgical objective in motor-eloquent tumour (MET) resection [2,3]. However, when CST is structurally compromised due to the presence of the tumour, the brain may recruit auxiliary networks to maintain functional output. We focus specifically on the basal ganglia motor loop, which modulates motor programme selection via cortico-striato-thalamo-cortical pathways, representing the most critical subcortical regulatory system capable of providing compensatory neuroplasticity to sustain corticomotor drive. The two ways that this paper will assess the motor network are via graph-theoretic connectome metrics and transcranial magnetic stimulation (TMS) generated metrics.

Graph-theoretic connectomics derived from diffusion MRI provides a quantitative framework for examining network topology, including degree, clustering coefficient, betweenness centrality, local efficiency, and small-worldness, beyond the description of individual tracts (please refer to Table 1 for understanding the meaning of the above terminologies) [4]. These metrics describe the organization of connections rather than just their magnitude, and there is increasing evidence that they are differentially affected by tumour biology, growth rate, and adaptive neuroplasticity [5].

Navigated transcranial magnetic stimulation (nTMS) provides complementary functional information such as localization (motor area identification) and function (motor system excitability) [6]. Cortical silent period (CSP) and resting motor threshold ratio (iRMTr) are indicators of intracortical inhibition and corticomotor excitability asymmetry, respectively [7]. Abnormal nTMS metrics have been associated with higher-grade gliomas and may be related to worse neurological outcomes after surgery [8].

Connectomics and nTMS, together, allow us to study the understudied structural network reorganization that is caused by the presence of adult diffuse gliomas. The 2021 WHO Classification stratifies adult diffuse gliomas principally by IDH mutational status: IDH-mutant tumours and IDH-wildtype glioblastoma [9]. Previous literature demonstrates that IDH-mutant gliomas, with their indolent growth, allow for compensatory neuroplasticity and preservation of small-world network efficiency compared to the rapid destruction seen in IDH-wildtype glioblastomas [10,11].

We hypothesize that differences in tumour growth kinetics and molecular patterns (specifically IDH mutational status) dictate distinct qualitative and quantitative patterns of structural network remodelling in the basal ganglia motor loop.

## 2. Methods

### 2.1. Participant Demographics and Clinical Assessment

Forty-eight consecutive adult patients with supratentorial METs were enrolled. Navigated transcranial magnetic stimulation (nTMS) mapping was performed preoperatively to locate the motor hotspot for the upper limb, as described in previous publications [12,13]. Neurophysiological metrics including the Cortical Excitability Score (CES), resting motor threshold (RMT) for the upper limb, its interhemispheric ratio (iRMTr) [14], and cortical silent period (CSP) were obtained. CES have been used to evaluate the disruption of functional areas (0 = normal, 1 = one functional area affected, 2 = both areas affected). The interhemispheric resting motor threshold ratio (iRMTr) of the upper limb evaluates how much magnetic stimulation is required to elicit a motor response. An increased iRMTr (>1.1) means the tumour-affected motor cortex is harder to excite than the healthy side, while an iRMTr below 0.9 indicates a decreased threshold. A 10% margin (0.9 to 1.1) was tolerated as normal [8].

**Figure 1:**
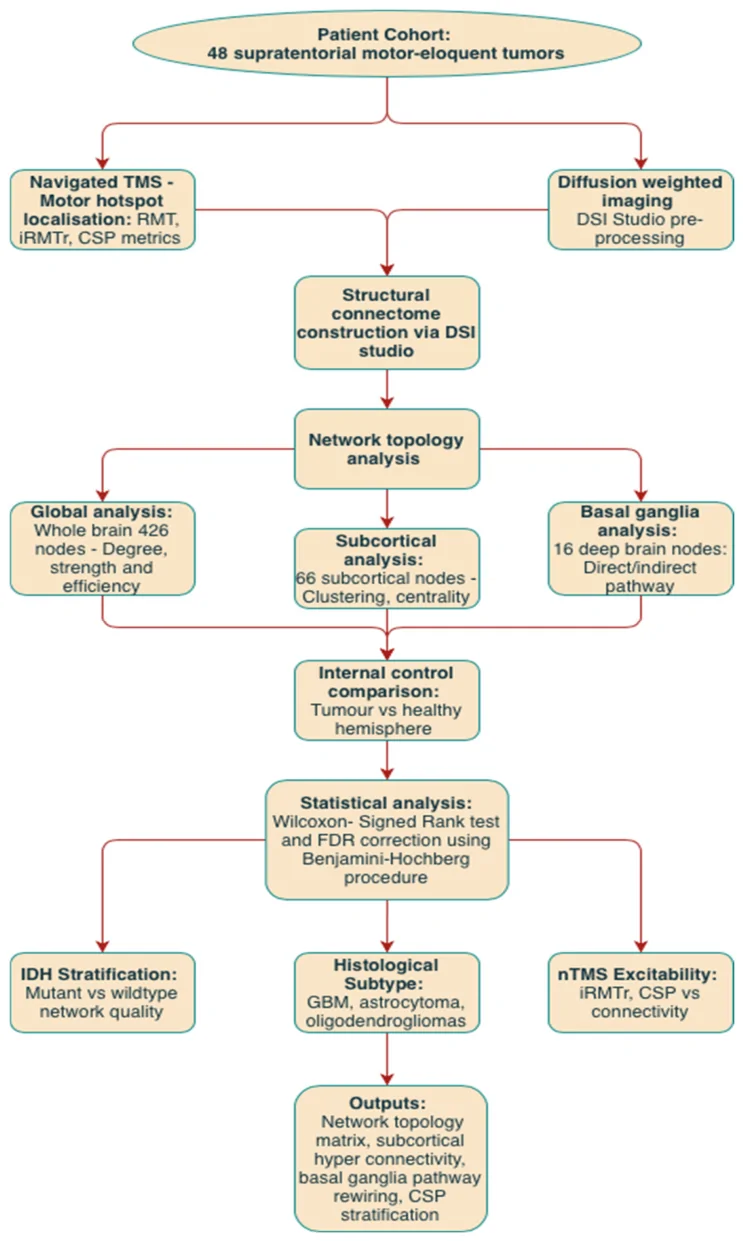
Overall methodology and results workflow.

The network assessed throughout this study was defined as an nTMS-seeded network for the motor hotspot of the upper limb. The main methodology is described below in Figure 1.

### 2.2. MRI Acquisition

All patients underwent preoperative MRI on a 3 Tesla scanner (Siemens Aera, Erlangen, Germany). The protocol included a high-resolution T1-weighted rapid gradient-echo (MPRAGE) (1 mm^3^ isotropic voxels, TR = 2300 ms, TE = 2.98 ms), a fluid-attenuated inversion recovery (FLAIR) sequence, and a T2-weighted sequence (1 mm^3^ isotropic voxels). Diffusion-weighted imaging used a single-shell protocol (TR: 9500 ms, TE: 86 ms, b = 1500 s/mm^2^, 64 non-collinear directions, plus three non-diffusion-weighted volumes, 2.5 mm isotropic voxels, anterior-to-posterior phase encoding). DSI employs q-space sampling to achieve superior angular resolution, enabling the resolution of complex fibre geometries. Structural connectomes were generated via DSI Studio^®^ 2025 using the HCPex Atlas (426 nodes). The connective matrices were generated utilizing streamline passing counts as the primary edge weight metric.

### 2.3. Diffusion MRI Preprocessing and Reconstruction

Raw diffusion-weighted imaging (DWI) data were preprocessed using the DSI Studio pipeline to ensure high data quality for subsequent tractography. Preprocessing included correction for susceptibility-induced distortions using FSL’s topup and for eddy current–induced distortions and subject motion using FSL’s eddy, as implemented within DSI Studio. Automated quality control procedures were applied to detect and exclude slices affected by signal dropout or excessive motion. The diffusion gradient table (b-table) was subsequently verified and corrected for proper spatial orientation. Following preprocessing, diffusion data were reconstructed using generalized q-sampling imaging (GQI), enabling improved resolution of complex fibre configurations, including crossing fibres, in subcortical white matter.

### 2.4. Co-Registration and Parcellation

T1 images were co-registered to the b0 volume of the diffusion data using a rigid-body transformation. To prevent topological artifacts secondary to the space-occupying lesion and peritumoural oedema, structural masking and cost-function masking techniques were employed during spatial normalization. Tumour volumes, including solid components and surrounding FLAIR-hyperintense oedema, were manually segmented on the T1-weighted MPRAGE and FLAIR sequences.

To parcellate the brain for network analysis, the HCPex 426-node atlas was utilized. Normalization between the subject’s native diffusion space and the standard MNI template was performed utilizing DSI Studio’s nonlinear diffeomorphic registration algorithm (QSDR). The overlapping of the HCPex parcels onto each subject’s native anatomy was manually inspected. Subjects exhibiting poor quality registrations, where the atlas parcels significantly mismatched the underlying cortical ribbons or deep grey matter boundaries due to uncompensated tumour volume, were discarded from the final connectomic analysis to prevent the introduction of topological artifacts.

### 2.5. Tractography and Connectome Construction

Whole-brain deterministic fibre tracking was performed in DSI Studio with the following parameters: angular threshold 60 degrees, step size half the voxel size (1.25 mm), QA termination threshold 0.05, smoothing 0.5, minimum streamline length 30 mm, maximum length 300 mm, one million seeds placed across the whole brain. Following the generation of the tractogram, structural connectivity matrices were generated for each subject using streamline counts as the primary edge weight. The motor hotspot identified on nTMS was projected onto the cortical surface and used to subset the global matrix: only nodes reachable from the hotspot (i.e., with at least one streamline traceable to the hotspot region) were retained, producing the upper-limb functionally informed connectome.

### 2.6. Subcortical and Basal Ganglia Network Analysis

The broader subcortical motor network, specifically the basal ganglia motor loop traversing the putamen, globus pallidus, substantia nigra, and ventrolateral thalamus, modulates motor programme selection via cortico-striato-thalamo-cortical pathways. To evaluate network alterations specifically within compensatory sub-circuits, these 66 subcortical nodes, and subsequently the 16 deep brain nodes that make up the basal ganglia motor loop, were isolated and analysed separately.

### 2.7. Topological Metrics

Six standard graph-theoretic metrics were computed for each network using the Brain Connectivity Toolbox in MATLAB (MATLAB_R2025a): degree, weighted strength, clustering coefficient, local efficiency, betweenness centrality, and PageRank centrality. The weighted clustering coefficient was the primary measure of local network organization [4].

### 2.8. TMS Metrics

TMS metrics such as CST volume, cortical silent period, and rest motor threshold were obtained and analysed in conjunction with the network metrics such as mean connections per node. Subanalysis was then carried out for each tumour subspecialty.

### 2.9. Statistical Analysis

All analyses were carried out in MATLAB. To determine the structural impact of the tumour, each patient’s own contralateral healthy hemisphere was used as an internal baseline control. Paired-sample *t*-tests (two-tailed) were employed to compare the mean network measures of the tumour hemisphere against the healthy hemisphere. Statistical significance was evaluated at an alpha level of *p* < 0.05. As this brain network data is rarely perfectly normally distributed, a Wilcoxon Signed-Rank test (using the signrank function in MATLAB) was performed.

Given the large number of topological metrics evaluated across different network scales, a False Discovery Rate (FDR) correction was applied to minimize Type I errors arising from multiple comparisons. The Benjamini–Hochberg procedure was utilized to adjust the generated *p*-values across all the distinct subanalyses. Specifically, FDR correction was applied independently to the graph-theoretical comparisons across the six topological metrics within the global cortical network, the 66-node subcortical network analysis, and the isolated 16-node basal ganglia motor loop analysis. Furthermore, the resulting *p*-values from the subgroup analyses stratifying the cohort by histological subtype (glioblastoma, astrocytoma, oligodendroglioma) and molecular phenotype (IDH-mutant versus IDH-wildtype) were also subjected to FDR correction. Only results with an FDR-adjusted *p*-value of < 0.05 were considered statistically significant.

## 3. Results

### 3.1. Demographics and Clinical Characteristics

Forty-eight patients were included (mean age 56.6 ± 14.9 years, range 27 to 78; 24 women and 24 men). All 48 patients were right-handed individuals as demonstrated in Table 2. Exclusion criteria included patients undergoing re-operations and biopsies alone. Tumours were left-hemispheric in 33 patients (68.7%) and right-hemispheric in 15. The primary histopathology included glioblastoma (IDH-wildtype, n = 19), oligodendroglioma (IDH-mutant 1p/19q-codeleted, n = 12), astrocytoma of various grades (IDH-mutant non-1p/19q-codeleted; n = 11), metastasis (n = 5), and radionecrosis/cyst (n = 1). The WHO grades included WHO Grade 4 (n = 22), WHO Grade 3 (n = 16), WHO Grade 2 (n = 3) and WHO Grade 1 (n = 1); the remaining six patients (five metastases and the radionecrosis case) were not gradable. None of the patients had any motor deficits pre- or postoperatively. 

### 3.2. nTMS Metrics

In total, 8 patients (16.7%) had a normal CES (score 0), 26 patients (54.2%) had an abnormal CES affecting one functional area (score 1), and no patients had an abnormal CES affecting both areas (score 2). Fourteen patients (29.1%) had indeterminate or missing CES data. In total, 14 patients (29.2%) had an abnormally increased iRMTr (>1.1) on the tumour side, 16 patients (33.3%) had an abnormally decreased iRMTr (<0.9), and 18 patients (37.5%) had an iRMTr within the normal range (between 0.9 and 1.1).

### 3.3. Global Network Topology

Baseline analysis of unaffected control hemispheres demonstrated that physical structural connectivity is highly symmetric between the left and right hemispheres. Despite the symmetry between the hemispheres, the healthy left cortex exhibited a significantly higher weighted betweenness centrality than the right cortex (*p* < 0.0001), in keeping with the established left-hemispheric bias in distributed cognitive hubs. Whole-brain network analysis demonstrated that the presence of the tumour did not significantly alter the global upper-limb informed connectome across any of the principal metrics (Supplementary Table S1). Although averages across the whole-brain network did not show significant variation, specific cortical nodes individually showed significant variations. Of the cortical nodes on the side of the tumour, significant hyper-connectivity was seen in the supplementary motor area (*p* = 0.0011), the premotor cortex (Area 6), with increased connection seen in 6 mp (medial premotor at *p* = 0.0033) and 6 d (dorsal premotor at *p* = 0.025) and the primary somatosensory cortex (*p* = 0.027).

### 3.4. Subcortical Network Alteration

**Figure 2:**
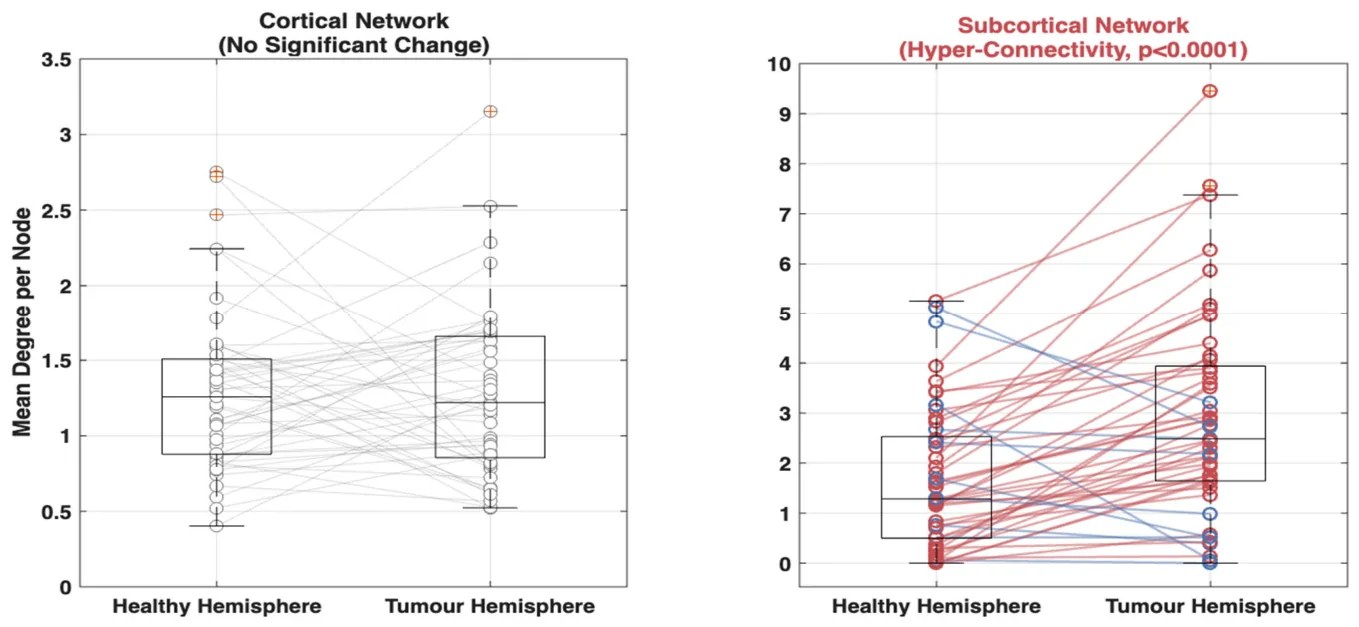
Overall cortical network (global cortical network) versus subcortical network divergence between the healthy and tumour hemispheres.

When analysis was restricted to the 66 subcortical nodes, the tumour hemisphere showed consistently higher connectivity metrics than the healthy hemisphere (Table 3), wherein left-sided tumoural involvement demonstrated higher degrees of significance across subcortical metrics compared to right-sided tumours, where degree and PageRank centrality did not reach statistical significance (*p* = 0.052 and *p* = 0.051, respectively). The graph below demonstrates the difference between the cortical and subcortical network alterations (Figure 2).

**Figure 3:**
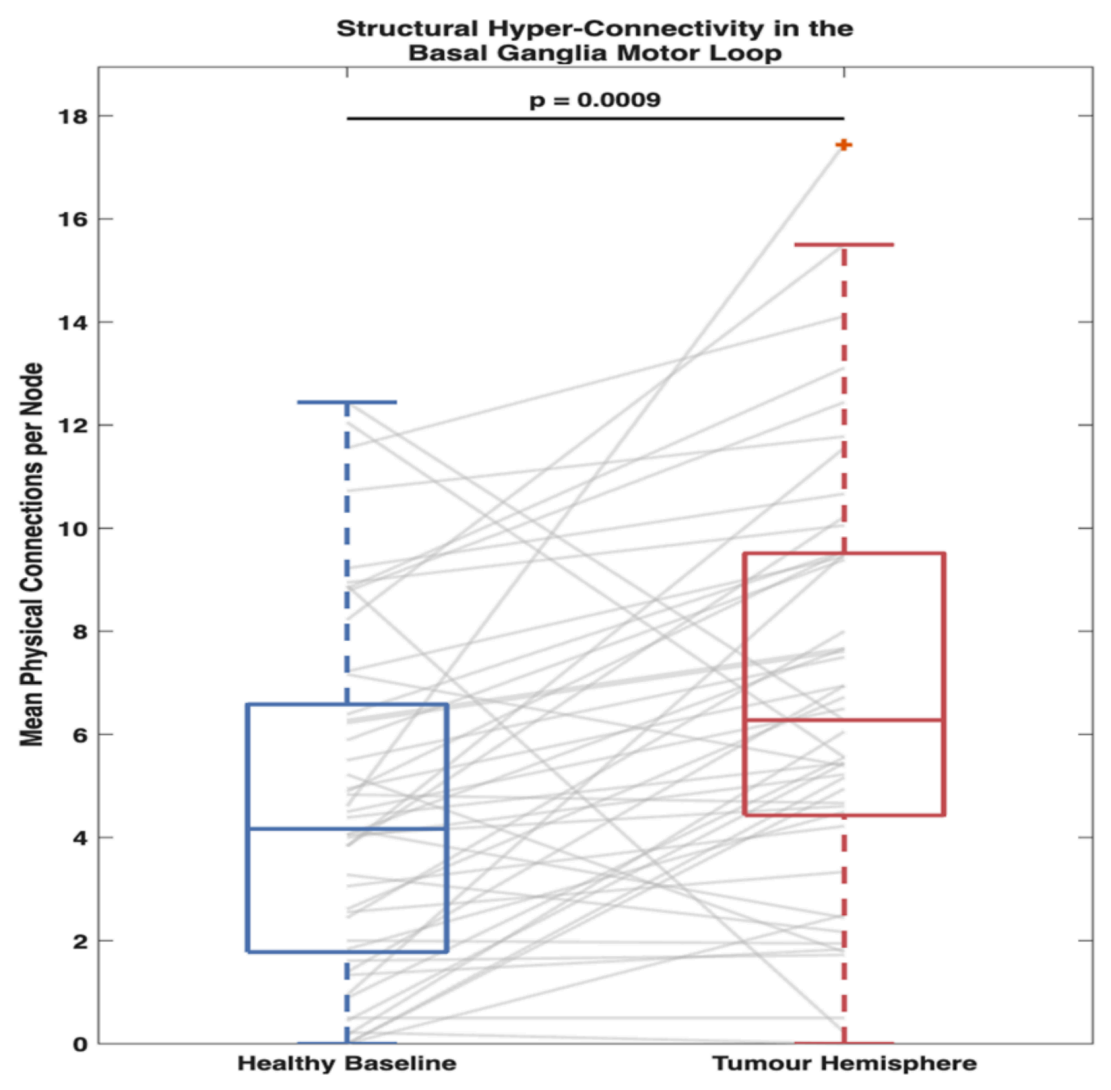
Structural hyper-connectivity specific to the basal ganglia motor loop—*p*= 0.0009.

To test our hypothesis, further subanalysis of the 16 deep brain nodes was done, comprising the basal ganglia motor loop, which confirmed significant structural differences in the tumour hemisphere, with mean connections on the tumour side (7.368) being significantly greater than the mean connections on the healthy side (5.186), with a *p*-value of 0.0009 (Table 4 and Figure 3).

**Figure 4:**
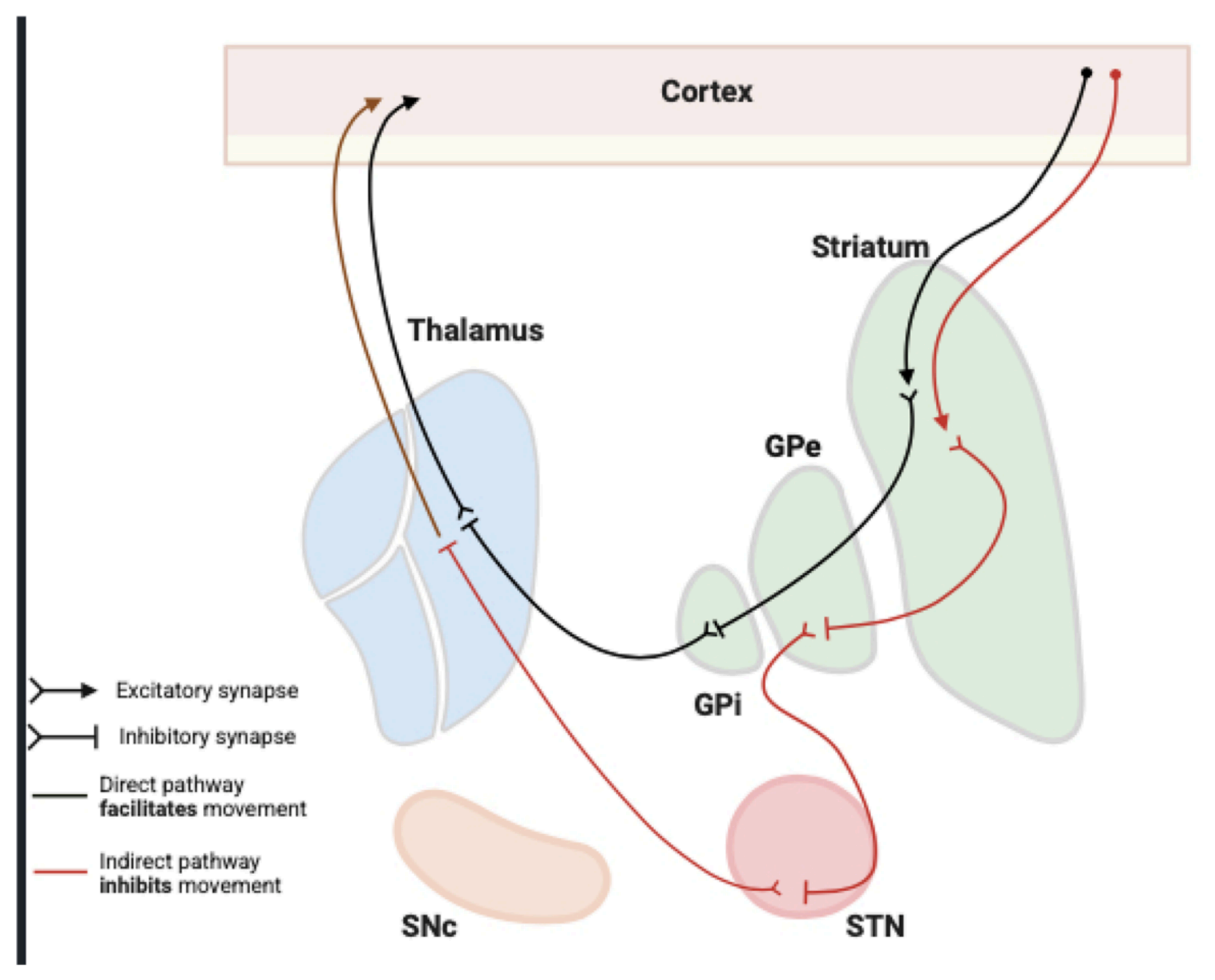
Pathway-specific structural reorganization within the basal ganglia motor loop (GPi and SNpr pathways show significant hyper-connectivity, *p* = 0.0009).

To understand the basal ganglia motor loop and the components of the motor loop, a further subanalysis was performed based on the diagram of the various components shown below (Figure 4).

Further subanalysis of the basal ganglia hyper-connectivity (Table 5) showed that there is a significant increase in the specific output nuclei of the direct pathway in the tumour hemisphere (GPi and SNpr; *p* = 0.0009) in comparison to the indirect pathway, which did not show any significant increase (*p* = 0.0882). The entry point of the signal from the cortex, the putamen, did not exhibit rewiring, but the output nuclei of the thalamus (*p* < 0.0001), prior to sending signals to the motor cortex, showed significant rewiring.

### 3.5. Network Organization by Histology

**Figure 5:**
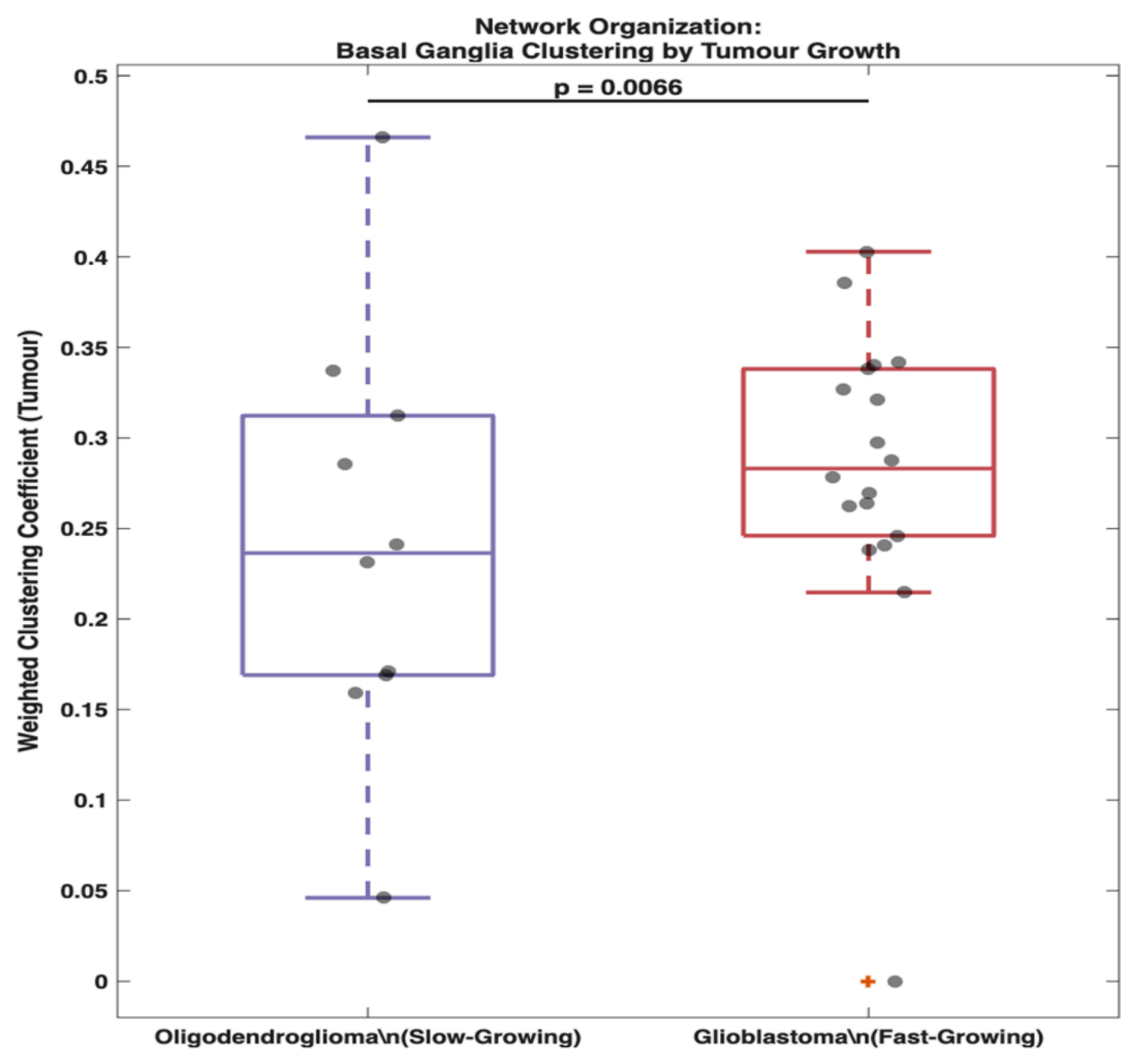
Basal ganglia motor loop clustering coefficient stratified by tumour growth velocity (oligodendroglioma vs. glioblastoma)—*p* = 0.0066.

Glioblastomas and oligodendrogliomas both showed significant subcortical and basal ganglia hyper-connectivity (*p* = 0.011 and *p* = 0.004, respectively). Astrocytomas showed an increase in subcortical hyper-connectivity but not significantly within the basal ganglia motor circuit. Within the basal ganglia loop, the weighted clustering coefficient differed between oligodendroglioma (mean 0.372) and glioblastoma (mean 0.274), with intermediate values for astrocytoma (0.331); the difference between oligodendroglioma and glioblastoma was significant for both clustering coefficient (*p* = 0.006) and local efficiency (*p* = 0.007) (Figure 5 and Table 5).

ANOVA revealed no significant difference in basal ganglia network clustering based on the lobar location of the tumour, indicating that the observed variations in topological clustering are driven by the tumour’s molecular biology rather than anatomical location.

### 3.6. Tumour Genetics and Network Plasticity

**Figure 6:**
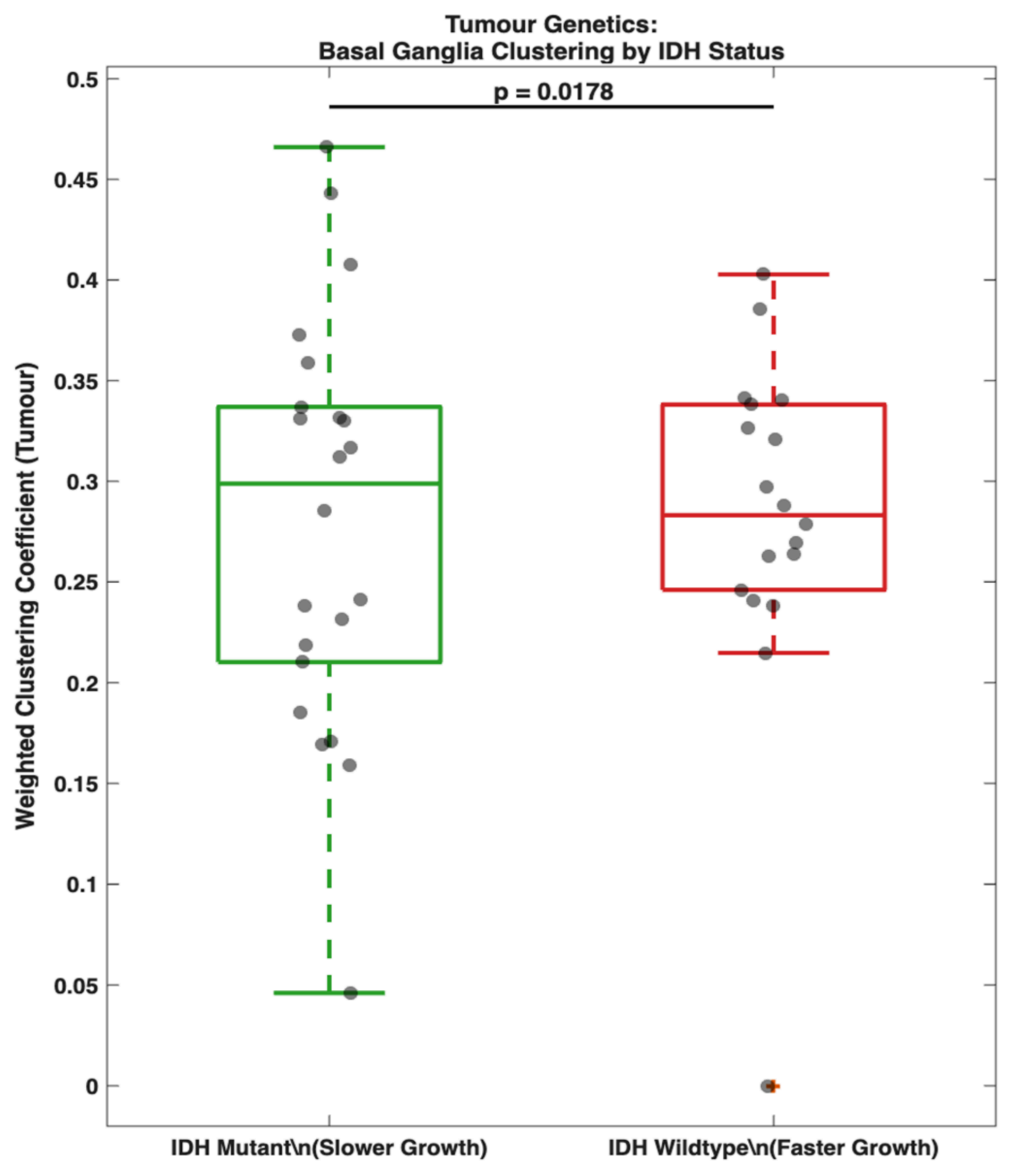
Network organization (clustering) differences based strictly on IDH mutational status—*p* = 0.0178.

The IDH-mutant cohort exhibited tightly clustered, highly organized local motor sub-circuits (0.345 ± SD 0.082), whereas the IDH-wildtype cohort exhibited significantly poorer network organization (0.273 ± SD 0.091; *p* = 0.0178), and this is demonstrated in Figure 6.

### 3.7. TMS Metrics and Connectivity

**Figure 7:**
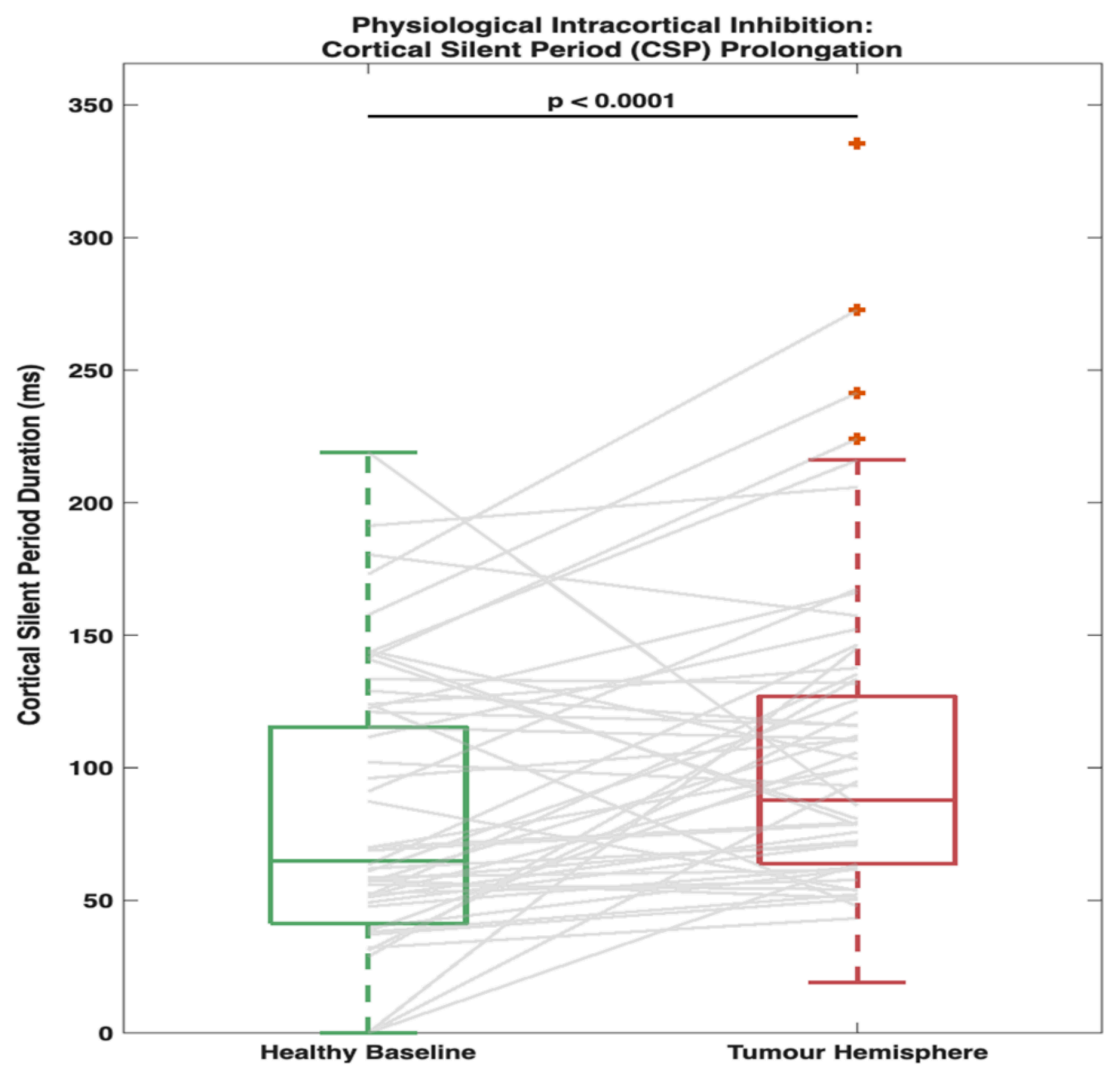
Prolongation of the cortical silent period (CSP) in the tumour hemisphere—*p* < 0.0001.

This study evaluated the impact of brain tumours on the motor system across three distinct modalities: macro-anatomy (CST volume), neurophysiology (TMS-derived CSP), and network topology (DSI structural connectivity). The presence of the tumour induced significant changes across two domains: loss of CST volume (*p* < 0.001) and prolongation of the cortical silent period indicative of intracortical inhibition (*p* < 0.0001). Figure 7 shows the graphical distribution of CSP in tumours versus healthy hemispheres.

Subanalysis based on histology revealed that the cortical silent period (CSP) was significantly prolonged in glioblastoma and oligodendrogliomas in the tumour hemisphere compared to astrocytomas, as shown in Table 6. There were not enough patients with metastasis in the analysis to determine significance.

Spearman correlations between CSP, iRMTr and the magnitude of basal ganglia hyper-connectivity were not significant (all *p* > 0.05). Likewise, neither tumour-side cortico-spinal tract volume nor fractional anisotropy correlated with the size of the direct-pathway or thalamic upregulation.

## 4. Discussion

This study describes how motor-eloquent tumours reshape the structural connectome anchored to the upper-limb motor hotspot. While traditional neurosurgical risk assessment focuses on the gross anatomical displacement of the cortico-spinal tract (CST), our findings demonstrate that functional adaptation occurs via targeted topological reorganization deep within the subcortex. Beneath a preserved global network topology, tumours induce substantial subcortical compensatory hyper-connectivity, particularly within the basal ganglia. This structural plasticity is not a random response to neoplasia, but the topological quality of this network reorganization is deeply stratified by the tumour’s molecular biology, specifically its IDH mutational status. A notable finding within the healthy hemisphere analysis was significant left-lateralization of betweenness centrality, consistent with left-hemispheric dominance in language and praxis hub networks [15], validating the sensitivity of the methodology.

This is consistent with principles of complex network resilience: a spatially circumscribed tumour represents a localized perturbation within a distributed, redundant network of hundreds of nodes, and global summary metrics are therefore insensitive to its presence [10]. This in turn implies that whole-brain average tractographic metrics are poor candidates to assess tumour-related impact on the connectome, as localized subcortical changes are obscured by averaging [16] and hence region-specific analysis is therefore essential.

Looking at tractographic metrics, co-elevation of degree, clustering and local efficiency suggests that new connections are integrated into clustered local sub-circuits rather than scattered randomly. This is consistent with directed reorganization of networks [2]. The concurrent increase in eigenvector centrality indicates that these hyperconnected nodes are preferentially attaching to other highly connected nodes, further reinforcing subcortical motor hub resilience [17,18]. In addition to the above, we have seen the hyper-connectivity in the supplementary motor area, premotor cortex and somatosensory cortex as well, indicating compensatory neuroplasticity [19]. It has been demonstrated that the sensorimotor network actively undergoes topological reorganization to participate in functional motor compensation in patients with gliomas [20].

Upon isolating the individual nodal components of the basal ganglia motor loop, we identified a highly pathway-specific compensatory mechanism. Structural hyper-connectivity in the tumour hemisphere was driven almost entirely by the selective upregulation of the excitatory direct pathway (GPi and SNpr) and the motor thalamic relay hubs, while the inhibitory indirect pathway (GPe) demonstrated no statistically significant topological alteration. This suggests that tumour-induced neuroplasticity does not trigger generalized subcortical sprouting but rather executes a targeted structural enhancement of the excitatory basal ganglia circuitry to preserve corticomotor drive when the primary CST is encroached by the tumour [21]. Interestingly, this structural excitatory drive contrasts with the significant prolongation of the cortical silent period (CSP) observed in glioblastomas and oligodendrogliomas, which indicates a simultaneous physiological increase in intracortical inhibition.

Basal ganglia clustering coefficient followed an ordering inversely related to tumour aggressiveness: oligodendroglioma (0.372) > astrocytoma (0.331) > glioblastoma (0.274) supporting the hypothesis that the time window afforded by slow tumour growth is a critical determinant of network adaptation quality [22]: indolent tumours allow the progressive formation of topologically efficient and tightly clustered motor sub-circuits [23], whilst the rapid, destructive growth of glioblastoma produces quantitatively high but qualitatively disorganized hyper-connectivity [22].

IDH stratification provided a molecularly precise dissection of the histological findings. Whilst IDH status did not significantly affect the absolute volume of subcortical hyper-connectivity, it exerted a significant effect on network organization quality [22]. This dissociation between connectivity quantity and topological quality fits with the broader observation that IDH-mutant tumours, with their slower growth, less perilesional oedema and a relatively preserved blood–brain barrier, create a microenvironment that is more permissive of effective axonal reorganization [24,25,26]. Clinically, preoperative knowledge of IDH status may inform expectations of residual motor network resilience following resection and guide peri-operative motor risk counselling [21]. With the rapid advancement of non-invasive preoperative molecular imaging techniques, such as radiogenomics, advanced MRI, and PET scanning [27], identifying the IDH mutation status prior to surgery [28] could reliably predict the patient’s baseline capacity for functional motor network reorganization.

From a clinical perspective, these network metrics offer insights into neuro-oncological outcomes. Our analysis revealed no significant difference in basal ganglia network clustering based on the lobar location or side of the tumour, indicating that the tumour’s molecular biology supersedes its gross anatomical topology in dictating large-scale network rewiring. The tightly clustered, highly organized local motor sub-circuits observed in indolent, IDH-mutant cohorts represent a new functional reserve in the presence of a tumour. Consequently, these connectomic markers hold substantial promise as non-invasive survival predictors. Integrating these specific topological metrics via multivariate analysis could eventually allow for the generation of ROC curves, creating predictive models to individualize peri-operative risk counselling and forecast both postoperative motor deficit trajectories and overall survival.

### Limitations and Future Directions

Several limitations must be acknowledged. The cohort size of 48 patients limits subgroup analyses, particularly for metastasis (n = 5) and for IDH-stratified comparisons. Second, single-shell GQI tractography is inherently susceptible to tumour-related oedema and susceptibility artefacts in perilesional tissue. While we attempted to overcome some of these limitations with the isolated healthy hemisphere internal control analysis, this paired design assumes baseline hemispheric connectivity symmetry, an assumption partially validated but not fully confirmed across all metrics. Future studies should aim to reproduce these findings with other algorithms for tractography, such as constrained spherical deconvolution or probabilistic algorithms, to ensure robustness. The reliance on a single tractography algorithm is a limitation, as different tracking methods can yield significantly variable connectomic reconstructions [29]. Validation by other research groups and the inclusion of TMS mapping for the lower limb, despite a historically lower success rate given the functional area’s location within the interhemispheric fissure [30], are also crucial next steps.

The reliance on a single deterministic tractography algorithm is a significant limitation, as different tracking methods (e.g., probabilistic vs. deterministic) and algorithms (e.g., constrained spherical deconvolution) can yield significantly variable connectomic reconstructions. While the use of the contralateral healthy hemisphere as an internal control is a robust strategy to mitigate inter-subject variability, the design assumes baseline hemispheric connectivity symmetry. While partially validated by findings in the healthy cohort, this assumption of perfect baseline symmetry is not universally confirmed across all topological metrics. Future efforts are required for the inclusion of nTMS mapping for the lower limb to complement the upper-limb data, particularly given the functional area’s challenging anatomical location within the interhemispheric fissure.

## 5. Conclusions

Motor-eloquent brain tumours drive a targeted reorganization of the functionally informed structural connectome that extends far beyond local anatomical displacement. The reorganization is concentrated in the excitatory direct pathway and motor thalamic relay, is mirrored by prolongation of the cortical silent period in glioblastoma and oligodendroglioma, and is shaped by the tumour’s molecular phenotype: IDH-mutant tumours are associated with more orderly compensatory networks than IDH-wildtype glioblastomas. nTMS-seeded structural connectomic profiling is feasible in routine preoperative planning and adds biologically interpretable information about the tumour-affected motor network.

Ultimately, integrating graph-theoretic connectomics with preoperative functional mapping offers a novel non-invasive framework in neuro-oncology. It not only deepens our understanding of tumour-induced hodotopical plasticity but may also provide clinicians with patient-specific network profiles to decode tumour aggressiveness, predict residual motor resilience, and individualize peri-operative surgical counselling.

## Figures and Tables

**Table 1:** Network terminologies.

Network Metric	General Meaning	Inference
**Degree**	The total number of links connected to a specific node in the brain	An increased **degree** means that there are extra tracts that are being/have been built
**Clustering coefficient**	The degree to which a node’s neighbours connect to each other (forming tightly knit groups)	**High clustering coefficient** indicates a tightly formed group of connections
**Betweenness Centrality**	The fraction of all the shortest paths in the network that pass through a given node	Nodes with **high centrality** indicate that they are important relay hubs
**Local efficiency**	Signal relay in the network despite a nodal disruption	**High local efficiency** indicates that even if a node is disrupted, signal relay continues unaffected
**Small worldness**	Combination metric of both clustering coefficient and betweenness centrality	Shows overall network resilience and efficiency in the presence of a tumour

Certain terminologies have been put in bold to ensure that the term stands out from the sentence and it is not to be confused with general terminology.

**Table 2:** Demographics.

WHO Grade	n	Age (Mean ± SD)	Gender (Male/Female)	Tumour Hemisphere (Left/Right)	Histopathological Diagnosis
Grade 2	3	49.7 ± 15.6	2/1	1/2	Astrocytoma (1) Oligodendroglioma (2)
Grade 3	16	50.0 ± 14.7	10/6	11/5	Astrocytoma (6) Oligodendroglioma (10)
Grade 4	22	60.3 ± 12.8	11/11	13/9	Glioblastoma (19) Astrocytoma (3)
Other Histology	7	62.0 ± 14.6	1/6	7/0	Metastasis (5) Astrocytoma (1) Other (1)
Total Cohort	48	56.5 ± 14.5	24/24	32/16	GBM (19), Astro (11), Oligo (12), Mets (5), Other (1)

**Table 3:** Subcortical network alterations (stratified by hemisphere).

Subcortical Topology	Tumour Side (Left, n = 33)	Healthy Side (Right, n = 33)	*p*-Value (Left)	Tumour Side (Right, n = 15)	Healthy Side (Left, n = 15)	*p*-Value (Right)
Degree (Binary)	2.897	1.584	0.0001 **	2.817	1.911	0.0516
Strength (Weighted)	2.335	1.337	0.0003 **	2.294	1.522	0.0353 **
Clustering Coef. (Weighted)	0.146	0.099	0.0008 **	0.139	0.097	0.0448 **
Local Efficiency (Weighted)	0.177	0.119	0.0004 **	0.168	0.117	0.0394 **
Eigenvector Cent. (Weighted)	0.031	0.019	<0.0001 **	0.028	0.020	0.0346 **
PageRank Cent. (Weighted)	0.0032	0.0026	<0.0001 **	0.0031	0.0026	0.0508

**—Indicates significant result as *p* < 0.05.

**Table 4:** Pathway-specific structural reorganization within the basal ganglia motor loop: mean degree refers to average number of connections for those specific anatomical nodes.

Basal Ganglia Sub-Circuit	Anatomical Nodes	Mean Degree (Healthy)	Mean Degree (Tumour)	*p*-Value
Shared Input Hub	Putamen	16.65	18.31	0.3100
Indirect Pathway	GPe, STN	9.84	12.30	0.0882
Direct Pathway	GPi, SNpr	4.93	7.76	0.0009 **
Shared Output Hub	Motor Thalamus	1.15	3.52	<0.0001 **

**—Indicates significant results with p value < 0.05.

**Table 5:** Network quality and histological grading.

Pathology/Histology	Clinical Growth Profile	Basal Ganglia Motor Loop Clustering	Network Quality
Oligodendroglioma	Indolent/Slow	0.372	Highly Organized/Efficient
Astrocytoma	Variable/Intermediate	0.331	Moderately Organized
Glioblastoma	Highly Aggressive/Fast	0.274	Low efficiency

**Table 6:** CSP prolongation and correlation with tumour subtype.

Histology Group	n	Mean CSP Healthy Side	Mean CSP Tumour Side	*p*-Value
Glioblastoma (WHO Grade 4)	19	81.1 ms	124.6 ms	0.0303
Oligodendroglioma (WHO Grade 2/3)	12	57.2 ms	99.0 ms	0.0028
Astrocytoma (WHO Grade 2/3/4)	11	93.9 ms	118.4 ms	0.0749
Metastasis	5	79.4 ms	75.8 ms	0.8246

## Data Availability

The data supporting the reported results can be requested from the corresponding author.

## Supplementary Materials

The following supporting information can be downloaded at https://www.mdpi.com/article/10.3390/cancers18182925/s1: Table S1: Global network topology (whole-brain average).
