# Supplementary material for: Functionally Informed Hand Knob Reveals Structural Connectome Differences in Motor-Eloquent Tumours
Source: Cancers (Basel). 2026 Sep 9;18(18):2925. doi: 10.3390/cancers18182925 (PMC13604273; doi:10.3390/cancers18182925)
Supplement: Supplementary file 1 [file cancers-18-02925-s001.zip › cancers-4385014-supplementary.pdf]

Supplementary

Table S1. Global Network Topology (Whole-Brain Average).

Network Measure	Mean (Tumour Hemisphere)	Mean (Healthy Hemisphere)	p-value
Degree (Binary)	1.530	1.367	0.121
Strength (Weighted)	1.171	1.074	0.243
Clustering Coef. (Weighted)	0.088	0.091	0.534
Local Efficiency (Weighted)	0.103	0.106	0.577
Betweenness Cent. (Weighted)	8.234	6.303	0.198
Small-World Index	0.903	0.903	0.884
